# Genomics by the beach

**DOI:** 10.1186/gb4171

**Published:** 2014-04-14

**Authors:** Peter F Hickey, Mark D Robinson

**Affiliations:** 1 Bioinformatics Division, Walter and Eliza Hall Institute of Medical Research, 1G Royal Parade, Parkville, Victoria 3052, Australia; 2 Department of Mathematics and Statistics, University of Melbourne, Parkville, Victoria 3010, Australia; 3 Institute of Molecular Life Sciences, University of Zurich, Winterthurerstrasse 190, Zurich 8057, Switzerland; 4 SIB Swiss Insitute of Bioinformatics, University of Zurich, Winterthurerstrasse 190, Zurich 8057, Switzerland

## Abstract

A report on the 35th Annual Lorne Genome Conference 2014 held in Lorne, Victoria, Australia, February 16–18, 2014.

## 

The 35th Annual Lorne Genome Conference attracted scientists from Australia and internationally to the Victorian Surf Coast town of Lorne for three days of genomics by the beach. The diversity of genomics research was showcased across 35 talks, including a session dedicated to young researchers and students, and more than 170 poster presentations.

The standard of scientific talks was extremely high in all sessions. However, in summarizing the meeting, we have chosen to focus on presentations that we can relate to most directly and that we found most interesting. Notably, only 15 of 35 talks were from Australian or New Zealand speakers, highlighting the variety of genomics talent, both in delegates and speakers, that remains the staple of the annual Lorne Genome meeting. The summertime setting, 40 meters from a popular beach, did little to dampen the spirits of the delegates.

## Genomes down under

Scientists have long been fascinated by Australia’s unique wildlife. In more recent times, scientists have studied the genomes of Australia’s marsupials and monotremes, such as the tammar wallaby and the platypus, to learn about embryonic development, sexual reproduction and the evolution of mammalian sex chromosomes. Genomics research has also been hugely important in the race to protect the Tasmanian devil from extinction in the wild due to Tasmanian devil facial tumor disease. Continuing in this tradition, Katherine Belov (University of Sydney, Australia) spoke about the antimicrobial and venom peptides of Australian mammals. The male platypus is one of the few venomous mammals, but only one amongst many of Australia’s other venomous creatures. Belov is studying the transcriptome and proteome of platypus venom to learn about its evolutionary origins. She also spoke about antimicrobial peptide genes found in marsupial and monotreme genomes, and presented evidence that these peptides are promising candidates as novel antibiotics against multi-drug-resistant bacterial strains. Will humans drink marsupial milk in the near future to ward off bugs?

With the world-renowned expert in Y chromosome biology, Professor Jenny Graves, in the audience, Henrik Kaessmann (Centre for Integrative Genomics, University of Lausanne, Switzerland) gave compelling insights into the evolution of Y chromosome and mammalian tissue transcriptomes. This is indeed a difficult challenge, since the Y chromosome is largely repetitive and refractory to short-read sequencing. Although not discussed, there is certainly scope for long-read third generation sequencing technologies to shine here and indeed, a quick search reveals various Twitter flurries on exactly this topic. Despite this, Henrik showed that, intriguingly, mutation rates are much higher for mammals and marsupials for the chromosome Y sequence compared with chromosome X sequence, yet this difference is absent from monotremes, such as the platypus.

Away from Australian-centric life forms, Jason Wong (University of New South Wales, Australia) delved into the growing field of proteogenomics, showing a data-based interplay between variants observed in RNA sequencing (RNA-seq) studies and their corresponding products in mass-spectrometry-based proteomics.

## Disease and medical genomics

Elaine Mardis (The Genome Institute at Washington University, USA) gave an engaging keynote presentation on translating cancer genomics research into clinical care. Mardis described how her team integrates whole genome sequencing (WGS), whole exome sequencing (WES) and RNA-seq. She highlighted that WGS and WES can be used to cross-validate each other, but even then, researchers often encounter an ongoing near-impossible data interpretation problem: long lists of mutations. In particular, she emphasized the extra information that RNA-seq can provide, which sometimes manifests in unusual ways. In one example, 40% of mutated genes are still expressed in the tumor. In another example, interpreting the long list of mutations was daunting, but it was spotted that FLT3 was wildly active in leukemic cells, which eventually led to the successful off-label use of Sunitinib. Aside from the challenges of integrating multiple data sources - data collection is easy, data interpretation is hard - Mardis also described how these results are then presented to the Washington University School of Medicine’s Tumor Board to help determine the clinical care of the patients. It is critical that physicians are well trained in understanding the strengths and limitations of genomics research, Mardis told the audience, and described three cases presented to the review board. Not only were these fascinating examples of translating cancer genomics into clinical practice, but they also drew shocked laughs from the mostly Australian audience about the realities of the American healthcare system.

## Epigenomics and genome organization

Epigenomics and the higher-order organization of the genome were the theme of several presentations. Bing Ren (University of San Diego School of Medicine, USA) spoke about detecting enhancers and reconstructing the three-dimensional structure of the genome using the chromatin conformation capture technology, Hi-C. Ren showed that Hi-C has more strings to its proverbial bow, and described his group’s recent foray into using Hi-C for long-range haplotyping of human and mouse genomes. Resolving haplotypes is still a difficult problem, particularly in mammalian genomes where most sequencing is done using Illumina’s short read technology. Haplotyping by Hi-C, along with recent improvements in throughput by Pacific Biosciences’ longer read technology and Illumina’s commercialization of the Moleculo technology, are helping to close the gap. In addition to phasing, Hi-C offers considerable insights into three-dimensional chromatin structure, allele-specific enhancer activity and their associations with allele-specific histone modifications and gene expression.

Wendy Bickmore (University of Edinburgh, UK) presented data showing that the most active regions of genomes are often found in the center of the nucleus, whereas the nuclear periphery is occupied by gene-poor, inactive chromosomal domains. Bickmore described recent experiments that seek to determine whether it is the genomic structure that dictates function or the function that dictates the structure by perturbing either the nuclear organization or transcription.

Joseph Ecker (Salk Institute, USA) closed the conference with a talk that took us from the world of human genomes to that of plants and back again through the common theme of DNA methylation. Ecker’s lab published some of the first genome-wide maps of DNA methylation in human cells and continues to do so as part of the NIH Epigenomics Roadmap consortium. It is well known that DNA methylation in mammalian cells mostly occurs at CpG dinucleotides. In contrast, non-CpG methylation is prevalent in plants, such as in the model organism *Arabidopsis*. Ecker presented work from his group and others that showed non-CpG methylation also exists in mammalian cells and can be widespread, particularly in pluripotent cell lines and in neurons. Ecker highlighted the power of model organisms by studying epialleles in inbred lines of *Arabidopsis*. Epialleles are genetically identical sequences with stable methylation patterns that can result in phenotypic variation. The effect of epialleles, even their very existence, are difficult to study in humans due to genetic variability. However, by careful breeding of *Arabidopsis* strains, Ecker’s team were able to remove the genetic variation and focus on the phenotypic variation driven by differences in DNA methylation patterns.

## Conclusions

Aside from the strong program of talks, the two poster sessions were very well attended and provided an excellent opportunity to meet new colleagues and catch up with old friends. Indeed, many discussions that started during the poster sessions were continued at the bar afterwards.

Another important question is how well did Lorne Genome get the gender balance? Overall, 43% of the talks were given by women, which is a great result given the corresponding ratio at the senior scientist level. Notably, there is room for improvement in the Millennium Science Young Investigators Award, now with 14 of 15 awards to men. Women dominated the poster prizes this year. Interestingly, the Lorne Protein Conference, one of the sister meetings to Lorne Genome, has been fully transparent since 2012 and publishes the gender ratios of abstract authors, selected abstracts, committee members, speakers, chairs and delegates. So, next time you are asked to chair, speak or be on a program committee, insist on publishing the gender statistics. In addition, the Walter and Eliza Hall Institute of Medical Research is to be thanked for their ongoing support of the Parent’s Viewing Room. We hope to see this family-friendly model exported to conferences elsewhere.

## Abbreviations

RNA-seq: RNA sequencing; WES: Whole exome sequencing; WGS: Whole genome sequencing.

## Competing interests

The authors declare that they have no competing interests.

## Author’s contributions

Both authors drafted the text, and read and approved the final manuscript.

